# Pheophorbide *b* ethyl ester from a *chlorella vulgaris* dietary supplement

**DOI:** 10.1107/S160053680802970X

**Published:** 2008-09-24

**Authors:** Chin Fei Chee, Noorsaadah Abdul Rahman, Sharifuddin M. Zain, Seik Weng Ng

**Affiliations:** a Department of Chemistry, University of Malaya, 50603 Kuala Lumpur, Malaysia

## Abstract

In the title compound, C_37_H_38_N_4_O_6_, four five-membered nitro­gen-bearing rings are nearly coplanar. Two N atoms in two these five-membered rings have attached H atoms, which contribute to the formation of intra­molecular N—H⋯N hydrogen bonds [N⋯N = 2.713 (5)–3.033 (6) Å].

## Related literature

For the crystal structure of pheophorbide *a* methyl ester, see: Fischer *et al.* (1972 ▶). For another example of a chlorin, see: Senge & Smith (1997 ▶).
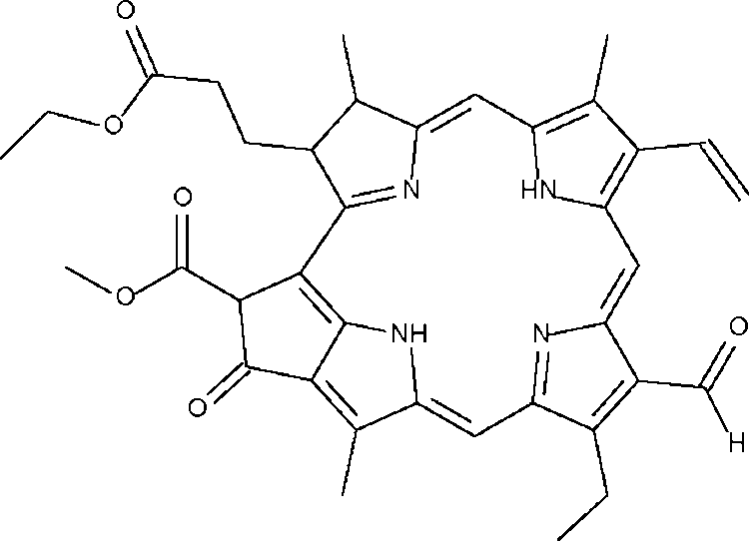


## Experimental

### 

#### Crystal data

C_37_H_38_N_4_O_6_
*M* *_r_* = 634.71
Monoclinic, 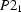

*a* = 7.0222 (2) Å
*b* = 30.5501 (7) Å
*c* = 7.8539 (2) Å
β = 112.300 (2)°
*V* = 1558.87 (7) Å^3^
*Z* = 2
Mo *K*α radiation
μ = 0.09 mm^−1^
*T* = 100 (2) K
0.35 × 0.20 × 0.01 mm

#### Data collection

Bruker SMART APEX diffractometer
Absorption correction: none
14310 measured reflections
3633 independent reflections
3100 reflections with *I* > 2σ(*I*)
*R*_int_ = 0.054

#### Refinement

*R*[*F* ^2^ > 2σ(*F* ^2^)] = 0.064
*wR*(*F* ^2^) = 0.177
*S* = 1.17
3633 reflections
438 parameters
3 restraints
H atoms treated by a mixture of independent and constrained refinement
Δρ_max_ = 0.55 e Å^−3^
Δρ_min_ = −0.38 e Å^−3^

### 

Data collection: *APEX2* (Bruker, 2007 ▶); cell refinement: *SAINT* (Bruker, 2007 ▶); data reduction: *SAINT*; program(s) used to solve structure: *SHELXS97* (Sheldrick, 2008 ▶); program(s) used to refine structure: *SHELXL97* (Sheldrick, 2008 ▶); molecular graphics: *X-SEED* (Barbour, 2001 ▶); software used to prepare material for publication: *publCIF* (Westrip, 2008 ▶).

## Supplementary Material

Crystal structure: contains datablocks global, I. DOI: 10.1107/S160053680802970X/cv2447sup1.cif

Structure factors: contains datablocks I. DOI: 10.1107/S160053680802970X/cv2447Isup2.hkl

Additional supplementary materials:  crystallographic information; 3D view; checkCIF report

## Figures and Tables

**Table 1:** Hydrogen-bond geometry (Å, °)

*D*—H⋯*A*	*D*—H	H⋯*A*	*D*⋯*A*	*D*—H⋯*A*
N1—H1N⋯N2	0.88 (3)	2.48 (9)	2.915 (6)	111 (7)
N1—H1N⋯N4	0.88 (3)	2.42 (8)	3.033 (6)	127 (8)
N3—H3N⋯N4	0.88 (3)	1.99 (4)	2.713 (5)	139 (5)

**Fig. 1.**
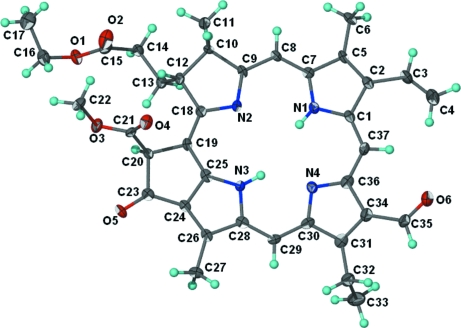
The molecular structure of the title compound showing the atomic numbering and 70% probability displacement ellipsoids.

## supplementary crystallographic information

### Experimental

A sample of the dietary supplement Chlorenergy (C'est Si Bon Co., USA) (20 g) was dissolved in 100 ml e thanol and 10 ml dilute sulfuric acid (10%, *v*/*v*). The mixture was heated at 323 K for 2 h. The solution was cooled and then stirred at room temperature for 3 days under normal day light (light intensity not measured), during which the solution turned green yellowish. The solvent was evaporated to dryness at reduced pressure. The crude product was purified by HPLC on an RP18e semi-preparative column (Merck Chromolith 100–10 mm) with acetonitrile-water (90:10, *v*/*v*) as the eluent. Pheophorbide *b* ethyl ester was isolated as brownish plates. Single crystals were grown by slow evaporation of a mixture of acetone and chloroform solution.

### Refinement

C-bound H-atoms were placed in calculated positions (C—H 0.95 to 0.99 Å) and were included in the refinement in the riding model approximation, with *U*_iso_(H) set to 1.2–1.5*U*_eq_(C). The amino H-atoms were located on a difference Fourier map, and were isotropically refined with a distance restraint N–H=0.88 (2) Å). In the absence of heavy scatterers, 3452 Friedel pairs were merged in the refinement.

### Crystal data

**Table:**

C_37_H_38_N_4_O_6_	*F*(000) = 672
*M**_r_* = 634.71	*D*_x_ = 1.352 Mg m^−^^3^
Monoclinic, *P*2_1_	Mo *K*α radiation, λ = 0.71073 Å
Hall symbol: P 2yb	Cell parameters from 3714 reflections
*a* = 7.0222 (2) Å	θ = 2.7–28.4°
*b* = 30.5501 (7) Å	µ = 0.09 mm^−^^1^
*c* = 7.8539 (2) Å	*T* = 100 K
β = 112.300 (2)°	Plate, brown
*V* = 1558.87 (7) Å^3^	0.35 × 0.20 × 0.01 mm
*Z* = 2	

### Data collection

**Table:**

Bruker SMART APEX diffractometer	3100 reflections with *I* > 2σ(*I*)
Radiation source: fine-focus sealed tube	*R*_int_ = 0.054
graphite	θ_max_ = 27.5°, θ_min_ = 1.3°
ω scans	*h* = −9→9
14310 measured reflections	*k* = −39→39
3633 independent reflections	*l* = −9→10

### Refinement

**Table:**

Refinement on *F*^2^	Primary atom site location: structure-invariant direct methods
Least-squares matrix: full	Secondary atom site location: difference Fourier map
*R*[*F*^2^ > 2σ(*F*^2^)] = 0.064	Hydrogen site location: inferred from neighbouring sites
*wR*(*F*^2^) = 0.177	H atoms treated by a mixture of independent and constrained refinement
*S* = 1.17	*w* = 1/[σ^2^(*F*_o_^2^) + (0.0986*P*)^2^ + 0.882*P*] where *P* = (*F*_o_^2^ + 2*F*_c_^2^)/3
3633 reflections	(Δ/σ)_max_ = 0.001
438 parameters	Δρ_max_ = 0.55 e Å^−^^3^
3 restraints	Δρ_min_ = −0.38 e Å^−^^3^

### Fractional atomic coordinates and isotropic or equivalent isotropic displacement parameters (Å^2^)

**Table:**

	*x*	*y*	*z*	*U*_iso_*/*U*_eq_	
O1	0.4731 (6)	0.50000 (12)	0.2630 (5)	0.0208 (8)	
O2	0.2600 (6)	0.49420 (13)	0.4146 (6)	0.0291 (9)	
O3	−0.0879 (6)	0.43359 (11)	0.0419 (5)	0.0197 (8)	
O4	−0.2243 (5)	0.39650 (12)	0.2153 (5)	0.0194 (7)	
O5	−0.1704 (5)	0.33907 (12)	−0.1938 (4)	0.0185 (7)	
O6	0.2153 (7)	0.07387 (13)	0.9416 (5)	0.0317 (9)	
N1	0.3166 (7)	0.25068 (14)	0.8896 (6)	0.0151 (8)	
H1N	0.236 (11)	0.251 (3)	0.772 (3)	0.06 (3)*	
N2	0.2567 (6)	0.32123 (13)	0.6242 (5)	0.0144 (8)	
N3	0.0540 (6)	0.25217 (13)	0.3274 (5)	0.0132 (8)	
H3N	0.101 (8)	0.2426 (19)	0.441 (3)	0.019 (15)*	
N4	0.1148 (6)	0.18688 (13)	0.5768 (5)	0.0145 (8)	
C1	0.3315 (7)	0.21173 (16)	0.9824 (6)	0.0136 (9)	
C2	0.4415 (7)	0.22289 (17)	1.1778 (6)	0.0147 (9)	
C3	0.4902 (9)	0.19315 (19)	1.3358 (7)	0.0270 (12)	
H3	0.5425	0.2079	1.4508	0.032*	
C4	0.4778 (11)	0.1527 (2)	1.3517 (8)	0.0388 (16)	
H4A	0.4273	0.1348	1.2451	0.047*	
H4B	0.5187	0.1399	1.4704	0.047*	
C5	0.4844 (7)	0.26621 (16)	1.1902 (6)	0.0149 (9)	
C6	0.6018 (8)	0.29141 (17)	1.3629 (6)	0.0173 (10)	
H6A	0.7268	0.2753	1.4360	0.026*	
H6B	0.5155	0.2952	1.4349	0.026*	
H6C	0.6395	0.3202	1.3303	0.026*	
C7	0.4064 (7)	0.28444 (16)	1.0051 (6)	0.0135 (9)	
C8	0.4182 (7)	0.32710 (16)	0.9564 (6)	0.0129 (9)	
H8	0.4804	0.3471	1.0545	0.016*	
C9	0.3481 (7)	0.34462 (16)	0.7770 (6)	0.0142 (9)	
C10	0.3693 (7)	0.39297 (15)	0.7423 (6)	0.0132 (9)	
H10	0.5172	0.4018	0.8000	0.016*	
C11	0.2387 (9)	0.42253 (16)	0.8135 (7)	0.0200 (10)	
H11A	0.2931	0.4215	0.9482	0.030*	
H11B	0.0959	0.4122	0.7647	0.030*	
H11C	0.2439	0.4527	0.7729	0.030*	
C12	0.2977 (7)	0.39420 (16)	0.5286 (6)	0.0137 (9)	
H12	0.1865	0.4165	0.4753	0.016*	
C13	0.4771 (7)	0.40329 (15)	0.4667 (6)	0.0151 (9)	
H13A	0.5884	0.3820	0.5277	0.018*	
H13B	0.4289	0.3983	0.3324	0.018*	
C14	0.5668 (8)	0.44977 (16)	0.5091 (7)	0.0187 (10)	
H14A	0.6030	0.4562	0.6413	0.022*	
H14B	0.6944	0.4514	0.4839	0.022*	
C15	0.4162 (8)	0.48372 (16)	0.3950 (7)	0.0180 (10)	
C16	0.3316 (9)	0.53099 (19)	0.1365 (7)	0.0243 (11)	
H16A	0.1884	0.5210	0.1061	0.029*	
H16B	0.3571	0.5320	0.0209	0.029*	
C17	0.3570 (9)	0.5764 (2)	0.2186 (9)	0.0294 (12)	
H17A	0.2695	0.5970	0.1262	0.044*	
H17B	0.5011	0.5855	0.2576	0.044*	
H17C	0.3167	0.5762	0.3252	0.044*	
C18	0.2128 (7)	0.34861 (15)	0.4764 (6)	0.0137 (9)	
C19	0.1041 (7)	0.33397 (15)	0.3001 (6)	0.0139 (9)	
C20	0.0327 (7)	0.36154 (15)	0.1264 (6)	0.0131 (9)	
H20	0.1544	0.3737	0.1058	0.016*	
C21	−0.1089 (7)	0.39854 (15)	0.1341 (6)	0.0135 (9)	
C22	−0.2130 (8)	0.47095 (16)	0.0499 (7)	0.0200 (10)	
H22A	−0.1934	0.4948	−0.0251	0.030*	
H22B	−0.1714	0.4807	0.1777	0.030*	
H22C	−0.3583	0.4624	0.0024	0.030*	
C23	−0.0867 (7)	0.32839 (16)	−0.0327 (7)	0.0158 (9)	
C24	−0.0692 (7)	0.28555 (15)	0.0547 (6)	0.0128 (9)	
C25	0.0404 (7)	0.29118 (16)	0.2478 (7)	0.0147 (9)	
C26	−0.1222 (7)	0.24197 (16)	0.0190 (6)	0.0139 (9)	
C27	−0.2368 (8)	0.22101 (17)	−0.1630 (7)	0.0191 (10)	
H27A	−0.3305	0.2424	−0.2461	0.029*	
H27B	−0.3163	0.1961	−0.1465	0.029*	
H27C	−0.1389	0.2107	−0.2158	0.029*	
C28	−0.0448 (7)	0.22063 (16)	0.1955 (6)	0.0145 (9)	
C29	−0.0575 (7)	0.17779 (16)	0.2429 (7)	0.0143 (9)	
H29	−0.1221	0.1577	0.1458	0.017*	
C30	0.0173 (7)	0.16128 (16)	0.4234 (7)	0.0157 (9)	
C31	0.0040 (7)	0.11672 (16)	0.4739 (7)	0.0180 (10)	
C32	−0.0871 (8)	0.07875 (17)	0.3481 (7)	0.0217 (10)	
H32A	−0.1830	0.0899	0.2278	0.026*	
H32B	−0.1672	0.0605	0.4011	0.026*	
C33	0.0764 (9)	0.0507 (2)	0.3185 (9)	0.0298 (12)	
H33A	0.0127	0.0240	0.2519	0.045*	
H33B	0.1821	0.0428	0.4380	0.045*	
H33C	0.1397	0.0671	0.2465	0.045*	
C34	0.0948 (8)	0.11562 (17)	0.6626 (7)	0.0207 (11)	
C35	0.1192 (9)	0.07653 (17)	0.7743 (7)	0.0216 (10)	
H35	0.0550	0.0506	0.7124	0.026*	
C36	0.1628 (8)	0.15993 (17)	0.7220 (7)	0.0177 (10)	
C37	0.2647 (8)	0.17179 (16)	0.9098 (7)	0.0194 (10)	
H37	0.2893	0.1486	0.9960	0.023*	

### Atomic displacement parameters (Å^2^)

**Table:**

	*U*^11^	*U*^22^	*U*^33^	*U*^12^	*U*^13^	*U*^23^
O1	0.0238 (18)	0.0243 (19)	0.0161 (17)	0.0037 (15)	0.0096 (14)	0.0056 (14)
O2	0.027 (2)	0.025 (2)	0.041 (2)	0.0052 (16)	0.0194 (18)	0.0084 (17)
O3	0.0251 (19)	0.0137 (17)	0.0232 (19)	0.0047 (14)	0.0125 (15)	0.0038 (14)
O4	0.0182 (16)	0.0191 (18)	0.0251 (18)	0.0012 (14)	0.0130 (14)	0.0029 (14)
O5	0.0202 (17)	0.0240 (19)	0.0094 (16)	0.0010 (14)	0.0034 (13)	0.0023 (13)
O6	0.052 (3)	0.021 (2)	0.023 (2)	−0.0026 (18)	0.0138 (18)	0.0042 (16)
N1	0.0177 (19)	0.0155 (19)	0.0128 (19)	0.0002 (15)	0.0068 (16)	0.0017 (15)
N2	0.017 (2)	0.012 (2)	0.014 (2)	−0.0004 (15)	0.0055 (16)	0.0021 (15)
N3	0.0161 (19)	0.0112 (19)	0.0125 (19)	0.0002 (15)	0.0058 (16)	0.0006 (15)
N4	0.0158 (19)	0.016 (2)	0.0136 (19)	−0.0003 (15)	0.0080 (16)	0.0009 (15)
C1	0.014 (2)	0.018 (2)	0.012 (2)	0.0036 (18)	0.0081 (17)	0.0018 (17)
C2	0.014 (2)	0.021 (2)	0.012 (2)	0.0024 (18)	0.0078 (18)	0.0013 (18)
C3	0.041 (3)	0.024 (3)	0.012 (2)	0.000 (2)	0.006 (2)	0.002 (2)
C4	0.053 (4)	0.032 (3)	0.018 (3)	−0.008 (3)	−0.002 (3)	0.006 (2)
C5	0.015 (2)	0.020 (2)	0.010 (2)	0.0031 (18)	0.0055 (18)	0.0038 (17)
C6	0.016 (2)	0.026 (3)	0.010 (2)	0.0034 (19)	0.0058 (18)	−0.0005 (18)
C7	0.011 (2)	0.016 (2)	0.014 (2)	0.0004 (17)	0.0057 (17)	−0.0003 (17)
C8	0.011 (2)	0.018 (2)	0.009 (2)	−0.0013 (17)	0.0034 (16)	0.0002 (17)
C9	0.013 (2)	0.018 (2)	0.011 (2)	0.0022 (18)	0.0044 (17)	0.0031 (17)
C10	0.017 (2)	0.013 (2)	0.009 (2)	−0.0014 (18)	0.0048 (17)	−0.0019 (17)
C11	0.032 (3)	0.014 (2)	0.018 (2)	0.002 (2)	0.014 (2)	0.0001 (18)
C12	0.016 (2)	0.013 (2)	0.014 (2)	0.0024 (17)	0.0089 (17)	0.0027 (17)
C13	0.017 (2)	0.015 (2)	0.016 (2)	0.0011 (18)	0.0088 (18)	0.0022 (17)
C14	0.023 (3)	0.018 (2)	0.013 (2)	−0.0040 (19)	0.0049 (19)	0.0009 (18)
C15	0.018 (2)	0.015 (2)	0.023 (2)	−0.0048 (18)	0.0096 (19)	−0.0008 (19)
C16	0.024 (3)	0.027 (3)	0.020 (3)	0.007 (2)	0.007 (2)	0.007 (2)
C17	0.027 (3)	0.026 (3)	0.037 (3)	0.004 (2)	0.015 (2)	0.003 (2)
C18	0.014 (2)	0.014 (2)	0.014 (2)	0.0047 (17)	0.0060 (18)	0.0012 (17)
C19	0.015 (2)	0.015 (2)	0.013 (2)	0.0012 (17)	0.0065 (17)	0.0039 (17)
C20	0.015 (2)	0.015 (2)	0.010 (2)	0.0020 (18)	0.0054 (17)	0.0009 (16)
C21	0.016 (2)	0.009 (2)	0.016 (2)	−0.0018 (17)	0.0053 (17)	−0.0015 (17)
C22	0.022 (2)	0.010 (2)	0.028 (3)	0.0020 (18)	0.010 (2)	−0.0006 (19)
C23	0.014 (2)	0.018 (2)	0.016 (2)	0.0022 (18)	0.0062 (18)	−0.0001 (18)
C24	0.009 (2)	0.019 (2)	0.012 (2)	0.0030 (17)	0.0056 (17)	0.0004 (17)
C25	0.009 (2)	0.021 (2)	0.015 (2)	0.0041 (18)	0.0054 (17)	0.0026 (18)
C26	0.011 (2)	0.019 (2)	0.013 (2)	0.0066 (18)	0.0072 (18)	0.0017 (18)
C27	0.019 (2)	0.021 (3)	0.016 (2)	−0.002 (2)	0.0052 (19)	−0.0046 (19)
C28	0.009 (2)	0.019 (2)	0.015 (2)	0.0037 (17)	0.0051 (17)	−0.0012 (18)
C29	0.013 (2)	0.013 (2)	0.018 (2)	−0.0019 (17)	0.0075 (18)	−0.0042 (18)
C30	0.014 (2)	0.014 (2)	0.022 (2)	−0.0014 (17)	0.0100 (19)	−0.0038 (18)
C31	0.012 (2)	0.019 (2)	0.024 (3)	−0.0003 (18)	0.0080 (19)	0.001 (2)
C32	0.021 (2)	0.018 (3)	0.022 (3)	−0.001 (2)	0.004 (2)	0.000 (2)
C33	0.032 (3)	0.026 (3)	0.031 (3)	0.002 (2)	0.011 (2)	−0.007 (2)
C34	0.027 (3)	0.017 (3)	0.021 (2)	0.000 (2)	0.011 (2)	0.0016 (19)
C35	0.033 (3)	0.014 (2)	0.022 (2)	−0.005 (2)	0.015 (2)	0.0004 (19)
C36	0.016 (2)	0.016 (2)	0.023 (2)	0.0008 (18)	0.0107 (19)	−0.0003 (19)
C37	0.030 (3)	0.012 (2)	0.018 (2)	0.003 (2)	0.011 (2)	0.0032 (18)

### Geometric parameters (Å, °)

**Table:**

O1—C15	1.340 (6)	C13—H13A	0.9900
O1—C16	1.456 (6)	C13—H13B	0.9900
O2—C15	1.207 (6)	C14—C15	1.509 (7)
O3—C21	1.332 (6)	C14—H14A	0.9900
O3—C22	1.456 (6)	C14—H14B	0.9900
O4—C21	1.208 (6)	C16—C17	1.511 (8)
O5—C23	1.220 (6)	C16—H16A	0.9900
O6—C35	1.231 (7)	C16—H16B	0.9900
N1—C7	1.360 (6)	C17—H17A	0.9800
N1—C1	1.379 (6)	C17—H17B	0.9800
N1—H1N	0.88 (3)	C17—H17C	0.9800
N2—C9	1.333 (6)	C18—C19	1.378 (6)
N2—C18	1.368 (6)	C19—C25	1.392 (7)
N3—C25	1.332 (6)	C19—C20	1.518 (6)
N3—C28	1.392 (6)	C20—C21	1.522 (6)
N3—H3N	0.88 (3)	C20—C23	1.579 (6)
N4—C36	1.342 (6)	C20—H20	1.0000
N4—C30	1.380 (6)	C22—H22A	0.9800
C1—C37	1.353 (7)	C22—H22B	0.9800
C1—C2	1.472 (6)	C22—H22C	0.9800
C2—C5	1.352 (7)	C23—C24	1.461 (7)
C2—C3	1.470 (7)	C24—C26	1.382 (7)
C3—C4	1.248 (9)	C24—C25	1.427 (6)
C3—H3	0.9500	C26—C28	1.439 (6)
C4—H4A	0.9500	C26—C27	1.493 (7)
C4—H4B	0.9500	C27—H27A	0.9800
C5—C7	1.456 (6)	C27—H27B	0.9800
C5—C6	1.505 (7)	C27—H27C	0.9800
C6—H6A	0.9800	C28—C29	1.373 (7)
C6—H6B	0.9800	C29—C30	1.405 (7)
C6—H6C	0.9800	C29—H29	0.9500
C7—C8	1.370 (7)	C30—C31	1.431 (7)
C8—C9	1.410 (6)	C31—C34	1.373 (7)
C8—H8	0.9500	C31—C32	1.500 (7)
C9—C10	1.519 (7)	C32—C33	1.520 (8)
C10—C11	1.535 (7)	C32—H32A	0.9900
C10—C12	1.559 (6)	C32—H32B	0.9900
C10—H10	1.0000	C33—H33A	0.9800
C11—H11A	0.9800	C33—H33B	0.9800
C11—H11B	0.9800	C33—H33C	0.9800
C11—H11C	0.9800	C34—C36	1.452 (7)
C12—C18	1.509 (7)	C34—C35	1.453 (7)
C12—C13	1.538 (6)	C35—H35	0.9500
C12—H12	1.0000	C36—C37	1.420 (7)
C13—C14	1.538 (7)	C37—H37	0.9500
			
C15—O1—C16	116.3 (4)	C16—C17—H17B	109.5
C21—O3—C22	114.4 (4)	H17A—C17—H17B	109.5
C7—N1—C1	112.2 (4)	C16—C17—H17C	109.5
C7—N1—H1N	131 (6)	H17A—C17—H17C	109.5
C1—N1—H1N	116 (6)	H17B—C17—H17C	109.5
C9—N2—C18	108.2 (4)	N2—C18—C19	121.0 (4)
C25—N3—C28	110.1 (4)	N2—C18—C12	113.3 (4)
C25—N3—H3N	135 (4)	C19—C18—C12	125.7 (4)
C28—N3—H3N	115 (4)	C18—C19—C25	126.5 (4)
C36—N4—C30	106.0 (4)	C18—C19—C20	126.3 (4)
C37—C1—N1	127.6 (4)	C25—C19—C20	107.2 (4)
C37—C1—C2	127.5 (4)	C19—C20—C21	112.0 (4)
N1—C1—C2	104.8 (4)	C19—C20—C23	104.5 (4)
C5—C2—C3	124.8 (5)	C21—C20—C23	110.4 (4)
C5—C2—C1	108.4 (4)	C19—C20—H20	109.9
C3—C2—C1	126.8 (5)	C21—C20—H20	109.9
C4—C3—C2	133.9 (5)	C23—C20—H20	109.9
C4—C3—H3	113.0	O4—C21—O3	124.3 (4)
C2—C3—H3	113.0	O4—C21—C20	124.2 (4)
C3—C4—H4A	120.0	O3—C21—C20	111.5 (4)
C3—C4—H4B	120.0	O3—C22—H22A	109.5
H4A—C4—H4B	120.0	O3—C22—H22B	109.5
C2—C5—C7	108.3 (4)	H22A—C22—H22B	109.5
C2—C5—C6	126.6 (4)	O3—C22—H22C	109.5
C7—C5—C6	125.1 (4)	H22A—C22—H22C	109.5
C5—C6—H6A	109.5	H22B—C22—H22C	109.5
C5—C6—H6B	109.5	O5—C23—C24	130.4 (5)
H6A—C6—H6B	109.5	O5—C23—C20	123.4 (4)
C5—C6—H6C	109.5	C24—C23—C20	106.1 (4)
H6A—C6—H6C	109.5	C26—C24—C25	109.1 (4)
H6B—C6—H6C	109.5	C26—C24—C23	143.1 (4)
N1—C7—C8	126.7 (4)	C25—C24—C23	107.8 (4)
N1—C7—C5	106.2 (4)	N3—C25—C19	138.0 (5)
C8—C7—C5	127.1 (4)	N3—C25—C24	107.7 (4)
C7—C8—C9	127.2 (4)	C19—C25—C24	114.2 (4)
C7—C8—H8	116.4	C24—C26—C28	105.5 (4)
C9—C8—H8	116.4	C24—C26—C27	127.8 (4)
N2—C9—C8	124.2 (4)	C28—C26—C27	126.7 (4)
N2—C9—C10	114.0 (4)	C26—C27—H27A	109.5
C8—C9—C10	121.8 (4)	C26—C27—H27B	109.5
C9—C10—C11	113.2 (4)	H27A—C27—H27B	109.5
C9—C10—C12	101.7 (4)	C26—C27—H27C	109.5
C11—C10—C12	112.6 (4)	H27A—C27—H27C	109.5
C9—C10—H10	109.7	H27B—C27—H27C	109.5
C11—C10—H10	109.7	C29—C28—N3	121.6 (4)
C12—C10—H10	109.7	C29—C28—C26	130.8 (4)
C10—C11—H11A	109.5	N3—C28—C26	107.6 (4)
C10—C11—H11B	109.5	C28—C29—C30	125.3 (4)
H11A—C11—H11B	109.5	C28—C29—H29	117.3
C10—C11—H11C	109.5	C30—C29—H29	117.3
H11A—C11—H11C	109.5	N4—C30—C29	123.3 (4)
H11B—C11—H11C	109.5	N4—C30—C31	111.1 (4)
C18—C12—C13	111.6 (4)	C29—C30—C31	125.6 (4)
C18—C12—C10	101.7 (4)	C34—C31—C30	105.7 (4)
C13—C12—C10	112.1 (4)	C34—C31—C32	126.8 (5)
C18—C12—H12	110.4	C30—C31—C32	127.5 (5)
C13—C12—H12	110.4	C31—C32—C33	112.3 (4)
C10—C12—H12	110.4	C31—C32—H32A	109.1
C14—C13—C12	114.6 (4)	C33—C32—H32A	109.1
C14—C13—H13A	108.6	C31—C32—H32B	109.1
C12—C13—H13A	108.6	C33—C32—H32B	109.1
C14—C13—H13B	108.6	H32A—C32—H32B	107.9
C12—C13—H13B	108.6	C32—C33—H33A	109.5
H13A—C13—H13B	107.6	C32—C33—H33B	109.5
C15—C14—C13	111.7 (4)	H33A—C33—H33B	109.5
C15—C14—H14A	109.3	C32—C33—H33C	109.5
C13—C14—H14A	109.3	H33A—C33—H33C	109.5
C15—C14—H14B	109.3	H33B—C33—H33C	109.5
C13—C14—H14B	109.3	C31—C34—C36	106.6 (4)
H14A—C14—H14B	107.9	C31—C34—C35	124.9 (5)
O2—C15—O1	123.5 (5)	C36—C34—C35	128.5 (5)
O2—C15—C14	124.9 (5)	O6—C35—C34	126.2 (5)
O1—C15—C14	111.5 (4)	O6—C35—H35	116.9
O1—C16—C17	111.5 (5)	C34—C35—H35	116.9
O1—C16—H16A	109.3	N4—C36—C37	126.2 (5)
C17—C16—H16A	109.3	N4—C36—C34	110.6 (4)
O1—C16—H16B	109.3	C37—C36—C34	123.1 (5)
C17—C16—H16B	109.3	C1—C37—C36	128.7 (5)
H16A—C16—H16B	108.0	C1—C37—H37	115.7
C16—C17—H17A	109.5	C36—C37—H37	115.7
			
C7—N1—C1—C37	−177.9 (5)	C19—C20—C21—O3	147.9 (4)
C7—N1—C1—C2	0.6 (5)	C23—C20—C21—O3	−96.1 (4)
C37—C1—C2—C5	178.4 (5)	C19—C20—C23—O5	178.5 (4)
N1—C1—C2—C5	0.0 (5)	C21—C20—C23—O5	58.0 (6)
C37—C1—C2—C3	−4.1 (8)	C19—C20—C23—C24	−2.4 (4)
N1—C1—C2—C3	177.5 (5)	C21—C20—C23—C24	−122.9 (4)
C5—C2—C3—C4	−173.5 (7)	O5—C23—C24—C26	0.1 (10)
C1—C2—C3—C4	9.4 (11)	C20—C23—C24—C26	−178.9 (6)
C3—C2—C5—C7	−178.1 (4)	O5—C23—C24—C25	−178.6 (5)
C1—C2—C5—C7	−0.5 (5)	C20—C23—C24—C25	2.3 (5)
C3—C2—C5—C6	4.8 (8)	C28—N3—C25—C19	−179.8 (5)
C1—C2—C5—C6	−177.7 (4)	C28—N3—C25—C24	−0.9 (5)
C1—N1—C7—C8	179.9 (4)	C18—C19—C25—N3	−0.8 (9)
C1—N1—C7—C5	−0.9 (5)	C20—C19—C25—N3	178.8 (5)
C2—C5—C7—N1	0.9 (5)	C18—C19—C25—C24	−179.7 (4)
C6—C5—C7—N1	178.1 (4)	C20—C19—C25—C24	−0.1 (5)
C2—C5—C7—C8	−179.9 (4)	C26—C24—C25—N3	0.1 (5)
C6—C5—C7—C8	−2.7 (8)	C23—C24—C25—N3	179.3 (4)
N1—C7—C8—C9	−3.0 (8)	C26—C24—C25—C19	179.3 (4)
C5—C7—C8—C9	178.0 (4)	C23—C24—C25—C19	−1.5 (5)
C18—N2—C9—C8	−179.1 (4)	C25—C24—C26—C28	0.7 (5)
C18—N2—C9—C10	1.7 (5)	C23—C24—C26—C28	−178.1 (6)
C7—C8—C9—N2	0.3 (8)	C25—C24—C26—C27	−179.8 (4)
C7—C8—C9—C10	179.4 (4)	C23—C24—C26—C27	1.5 (9)
N2—C9—C10—C11	113.3 (4)	C25—N3—C28—C29	−178.2 (4)
C8—C9—C10—C11	−65.9 (6)	C25—N3—C28—C26	1.3 (5)
N2—C9—C10—C12	−7.8 (5)	C24—C26—C28—C29	178.3 (5)
C8—C9—C10—C12	173.0 (4)	C27—C26—C28—C29	−1.3 (8)
C9—C10—C12—C18	9.8 (4)	C24—C26—C28—N3	−1.2 (5)
C11—C10—C12—C18	−111.7 (4)	C27—C26—C28—N3	179.2 (4)
C9—C10—C12—C13	−109.5 (4)	N3—C28—C29—C30	2.5 (7)
C11—C10—C12—C13	129.0 (4)	C26—C28—C29—C30	−176.9 (4)
C18—C12—C13—C14	177.9 (4)	C36—N4—C30—C29	179.8 (4)
C10—C12—C13—C14	−68.7 (5)	C36—N4—C30—C31	−0.2 (5)
C12—C13—C14—C15	−68.1 (5)	C28—C29—C30—N4	−0.2 (7)
C16—O1—C15—O2	−1.7 (7)	C28—C29—C30—C31	179.8 (5)
C16—O1—C15—C14	176.1 (4)	N4—C30—C31—C34	0.4 (6)
C13—C14—C15—O2	70.4 (7)	C29—C30—C31—C34	−179.6 (5)
C13—C14—C15—O1	−107.3 (5)	N4—C30—C31—C32	−178.9 (5)
C15—O1—C16—C17	81.0 (6)	C29—C30—C31—C32	1.1 (8)
C9—N2—C18—C19	−175.3 (4)	C34—C31—C32—C33	−78.8 (7)
C9—N2—C18—C12	5.7 (5)	C30—C31—C32—C33	100.3 (6)
C13—C12—C18—N2	109.5 (4)	C30—C31—C34—C36	−0.4 (5)
C10—C12—C18—N2	−10.2 (5)	C32—C31—C34—C36	178.8 (5)
C13—C12—C18—C19	−69.4 (6)	C30—C31—C34—C35	−178.9 (5)
C10—C12—C18—C19	170.9 (4)	C32—C31—C34—C35	0.3 (9)
N2—C18—C19—C25	−5.0 (7)	C31—C34—C35—O6	173.3 (6)
C12—C18—C19—C25	173.9 (4)	C36—C34—C35—O6	−4.9 (9)
N2—C18—C19—C20	175.5 (4)	C30—N4—C36—C37	−179.8 (5)
C12—C18—C19—C20	−5.6 (7)	C30—N4—C36—C34	−0.1 (5)
C18—C19—C20—C21	−59.4 (6)	C31—C34—C36—N4	0.3 (6)
C25—C19—C20—C21	121.0 (4)	C35—C34—C36—N4	178.8 (5)
C18—C19—C20—C23	−178.9 (4)	C31—C34—C36—C37	−180.0 (5)
C25—C19—C20—C23	1.5 (5)	C35—C34—C36—C37	−1.5 (8)
C22—O3—C21—O4	1.7 (7)	N1—C1—C37—C36	−0.3 (9)
C22—O3—C21—C20	−177.6 (4)	C2—C1—C37—C36	−178.4 (5)
C19—C20—C21—O4	−31.4 (6)	N4—C36—C37—C1	1.1 (9)
C23—C20—C21—O4	84.6 (5)	C34—C36—C37—C1	−178.6 (5)

### Hydrogen-bond geometry (Å, °)

**Table:**

*D*—H···*A*	*D*—H	H···*A*	*D*···*A*	*D*—H···*A*
N1—H1N···N2	0.88 (3)	2.48 (9)	2.915 (6)	111 (7)
N1—H1N···N4	0.88 (3)	2.42 (8)	3.033 (6)	127 (8)
N3—H3N···N4	0.88 (3)	1.99 (4)	2.713 (5)	139 (5)
